# Village doctor-assisted case management of rural patients with schizophrenia: protocol for a cluster randomized control trial

**DOI:** 10.1186/1748-5908-9-13

**Published:** 2014-01-16

**Authors:** Wenjie Gong, Dong Xu, Liang Zhou, Henry Shelton Brown III, Kirk L Smith, Shuiyuan Xiao

**Affiliations:** 1 School of Public Health, Central South University, 110 Xiangya Road, Changsha 410078, Hunan, China; 2 Department of Global Health, University of Washington, Seattle, WA, USA; 3 UTHealth School of Public Health, Austin Regional Campus Michael & Susan Dell Center for Healthy Living, Austin, TX, USA; 4 Department of Internal Medicine, University of Texas Medical Branch, 301 University Boulevard, Galveston, TX, USA

**Keywords:** Schizophrenia case management, Village doctor, Community health worker, Cluster randomized controlled trial, Project 686, Rural community health, Drug compliance, Mental health intervention

## Abstract

**Background:**

Strict compliance with prescribed medication is the key to reducing relapses in schizophrenia. As villagers in China lack regular access to psychiatrists to supervise compliance, we propose to train village ‘doctors’ (*i.e*., villagers with basic medical training and currently operating in villages across China delivering basic clinical and preventive care) to manage rural patients with schizophrenia with respect to compliance and monitoring symptoms. We hypothesize that with the necessary training and proper oversight, village doctors can significantly improve drug compliance of villagers with schizophrenia.

**Methods/design:**

We will conduct a cluster randomized controlled trial in 40 villages in Liuyang, Hunan Province, China, home to approximately 400 patients with schizophrenia. Half of the villages will be randomized into the treatment group (village doctor, or VD model) wherein village doctors who have received training in a schizophrenia case management protocol will manage case records, supervise drug taking, educate patients and families on schizophrenia and its treatment, and monitor patients for signs of relapse in order to arrange prompt referral. The other 20 villages will be assigned to the control group (case as usual, or CAU model) wherein patients will be visited by psychiatrists every two months and receive free antipsychotic medications under an on-going government program, Project 686. These control patients will receive no other management or follow up from health workers. A baseline survey will be conducted before the intervention to gather data on patient’s socio-economic status, drug compliance history, and clinical and health outcome measures. Data will be re-collected 6 and 12 months into the intervention. A difference-in-difference regression model will be used to detect the program effect on drug compliance and other outcome measures. A cost-effectiveness analysis will also be conducted to compare the value of the VD model to that of the CAU group.

**Discussion/implications:**

Lack of specialists is a common problem in resource-scarce areas in China and other developing countries. The results of this experiment will provide high level evidence on the role of health workers with relatively limited medical training in managing severe psychiatric disease and other chronic conditions in developing countries.

**Trial registration:**

ChiCTR-TRC-13003263.

## Background

Schizophrenia affects approximately 0.3% to 0.7% of people at some point in their lives [1], or 24 million people worldwide [2], accounting for an estimated 1.1% of global DALYs (disability-adjusted life years) and 2.8% of global YLDs (years lived with disability) [3]. Approximately three-fourths of people with schizophrenia have ongoing disability due to relapses [4]. Compared with the general population, schizophrenia patients have significantly lower scores in physical and psychological QoL (Quality of Life) domains [5].

According to an epidemiological survey in four provinces in China during 2001 to 2005, the adjusted prevalence of schizophrenia is 7.81 per 1,000; more than half of the patients live in rural China with little or no access to professional psychiatrists [6]. Even when treatment is available to villagers, many remain unaware of, or face barriers in accessing, treatment.

### Drug compliance

Since chlorpromazine was introduced in 1952, antipsychotics have become the principal element of schizophrenia treatment, and compliance is key to reducing relapse. A number of studies outside of China have demonstrated that partial compliance with antipsychotic medication is a common problem and is associated with greater risk of hospitalization [7,8]. In a German study, ‘Needing someone to remind them to take their medication’ contributed to partial compliance in 62% of patients [9]. In a previous unpublished study conducted by our group, only 20.8% of villagers with schizophrenia in Liuyang Municipality were under any kind of supervision of their drug-taking, and less than 50% of schizophrenia patients took their daily medication over the past month, consistent with the results of a study in Qingpu, a municipality near Shanghai, which showed that 60% of patients fail to comply with prescribed treatments [10].

### Project 686

The ‘Management of Major Psychoses Treatment Project,’ funded by the central government and known as Project 686, aims to improve the treatment of severe psychoses by dispensing free medications in rural China, with psychiatric professionals visiting rural communities every two months to dispense antipsychotics to patients. Based on our preliminary data, among the 4,000 Project 686 patients registered in Liuyang Municipality, Hunan Province, as having severe mental disorders -- most of them schizophrenia patients -- only approximately 70% obtained their free antipsychotics in the previous two months, and for those who got medications, no information exists regarding actual use. Despite the availability of free medication, challenges to compliance persist; these include engaging family members in compliance, and the two-month interval between psychiatric visits and dispensation of medications [11].

### Case management and village doctors

Community support services commonly available to psychiatric patients in developed countries include drop-in centers, visits by a community mental health team, facilitated employment [9] and support groups. Intensive Case Management (ICM) is a long-term, community-based package of care for severely mentally ill people who do not require immediate admission. Studies comparing various models of ICM involving a variety of caregivers such as family members, mental health workers, psychiatrists-core teams, and community health workers have demonstrated the benefit of ICM for patients with schizophrenia [12,13], including schizophrenic patients in urban areas of China [14,15]; however, it has been difficult to scale up these models in rural China, mainly due to lack of human resources [16]. It is important to develop rational, targeted, and cost-effective risk-reduction strategies to address this human resources issue.

Evidence indicates that regular visits by psychiatrists have a positive effect on treatment compliance of patients with schizophrenia in rural China [11]. However, it is not feasible to depend solely on such specialists for case management given the large population size versus limited number of psychiatrists in rural China. Lay health counselors (LHCs) have been shown to be effective in the treatment of common mental disorders in low-and middle-income countries (LMICs) [17]. It is plausible that village ‘doctors’ are the most appropriate candidates to serve as LHCs in rural China. Village doctors (VDs), despite their title as ‘doctor,’ are individuals with very basic medical training who provide primary and preventive care to villagers in almost every village in China; because VDs are recruited from among the villagers themselves, they are highly conversant with village residents’ health conditions and concerns. Earlier research on the effects of directly observed chemotherapy on tuberculosis, conducted in 1991, showed that satisfactory treatment adherence and response rates were achieved using VDs as case managers [18].

### Implications of our experiment

This study will integrate a village doctor-assisted case management model into the existing Project 686 sites in rural communities of Liuyang. VDs will receive antipsychotics from psychiatrists every two months, store the medications, manage schizophrenia cases by dispensing medications to patients and their family members every week or every day (both intervention strategies will be tested), counsel patients and family members about the importance of compliance, address concerns raised by patients and family members, and monitor patients for signs of disease and indications of non-compliance. Using a cluster randomized trial (CRT), we will compare the effects of this VD model with the care-as-usual (CAU) model employed in Project 686 in which visiting psychiatrists dispense drugs to villagers every two months, but provide no other services. To the best of our knowledge, this is the first study using VDs as case managers for schizophrenia in China. The proposed study has important implications for improving the management of schizophrenia patients in over 100 communities enrolled in Project 686 in China, and will generate findings as to the effectiveness of involving low level health workers and family members in managing mental disorders common in rural China.

## Research design and methods

### Hypotheses and conceptual model

Hypothesis: Patients in the VD model will have higher rates of drug compliance and greater social function than those in the CAU model with respect to symptom relief, quality of life, hospitalization days and risky behaviors.

**Figure 1:**
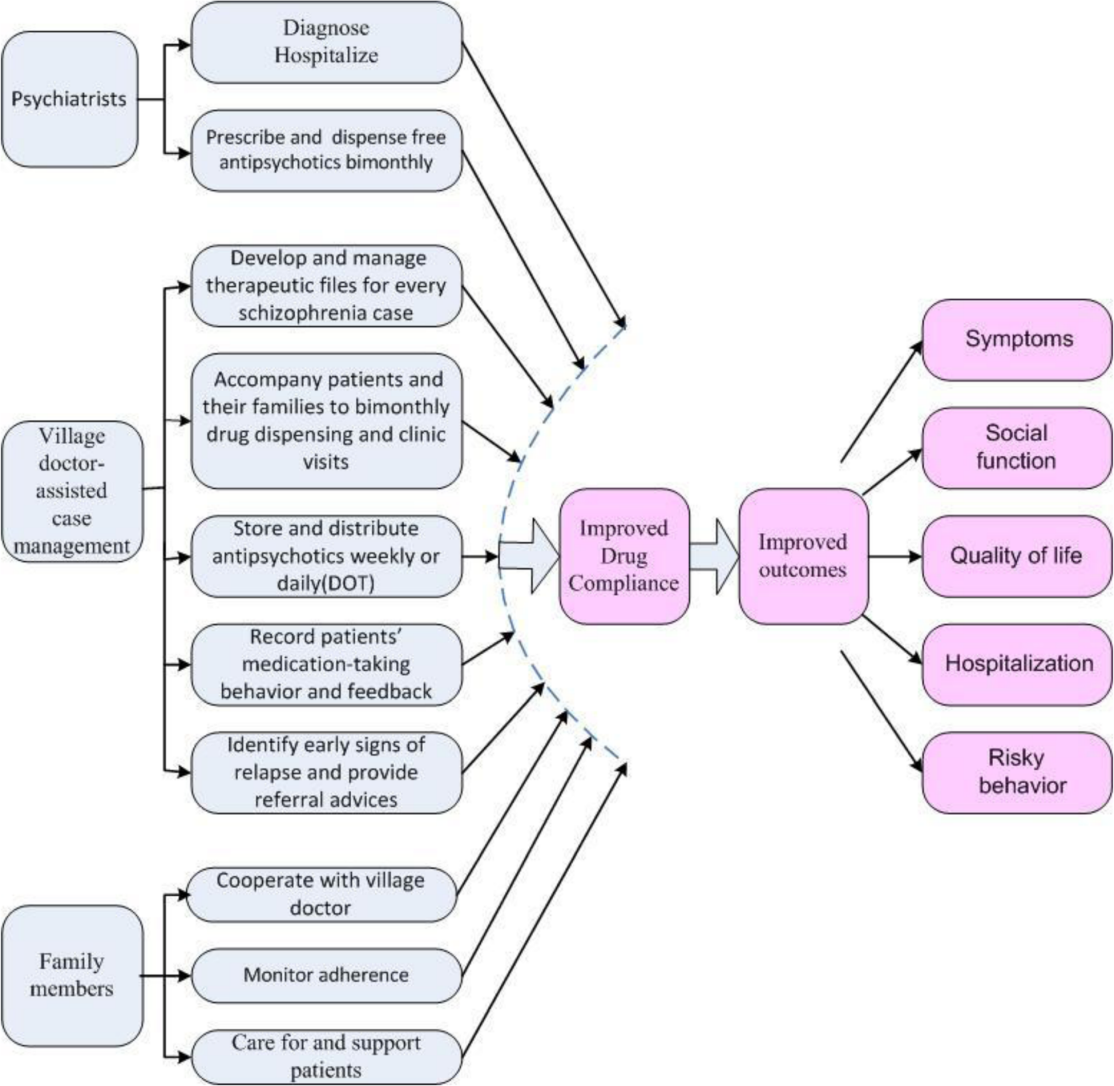
Conceptual model.

Conceptual model: Figure 1 illustrates the conceptual model. In this model, VDs exercise five functions of case management: maintaining patient files; accompanying patients to bimonthly psychiatrist visits; storing and distributing antipsychotics; monitoring drug-taking; and monitoring patients for symptoms.

## Methods

### Sampling and cluster randomized controlled design

We will conduct a cluster randomized controlled trial involving a sample of rural schizophrenia patients in Liuyang Municipality, Hunan Province, China. Criteria for the sample include:

1. Resided in one of the administrative rural villages of Liuyang Municipality for the past year.

2. Diagnosed by psychiatrist as having schizophrenia.

3. Registered for free medication under Project 686.

Due to our long time working relationship with the local government in Liuyang, they have agreed to assist us in the random selection of villages. A total of 40 administrative villages will be selected out of 318 administrative villages at random. We will use the statistical software package STATA to generate 40 random numbers. All villages will be randomized into two groups (the intervention arm and the control arm) by block sampling (block size = four). The randomization will be based on villages rather than individuals. Due to the nature of our experiment, the allocation will not be blinded for the service providers, patients and interviewers, but those who analyze the data will be blinded for the group information. The co-PI of this project, who is a statistician, will be responsible for the random allocation sequence and the assignment of clusters to interventions.

### Sample size

To minimize contamination and simplify project management, we will use one-stage clustered sampling. For a two-sided significance level of 0.05 and power of 80%, the sample size is calculated for both the binary variable Drug Compliance (DC) and the continuous variable Drug Compliance Score (DCS), with DC referring to a patient outcome achieving pre-defined compliance or not, while DCS refers to a score anywhere from 0 (no compliance) to 10 (full compliance), calculated as the proportion of the number of drugs actually taken by an individual divided by the number of drugs prescribed for that individual over a given period of time. As our previous study of Project 686 patients with schizophrenia in Liuyang Municipality showed a 50% compliance rate, or DC of 0.5, our calculations were based on the need to detect DC in the range of 0.5 (the current rate) to 0.7 (our hypothesized improvement). In conducting our sample size analysis, we used software developed by the University of Aberdeen, U.K., specifically for clustered randomized trials [19].

### Sample size with the binary variable drug compliance (DC)

For a cluster randomized trial, the sample size must be ‘inflated’ over that of an individual randomized controlled trial (IRCT), taking into consideration the size of the cluster as well as the intra-cluster correlation coefficient (ICC). As we are uncertain about the ICC and the effect size associated with our intervention, we did a further sensitivity analysis (as shown in Table 1) on the minimum effect size detectable in relation to various sample sizes and ICCs, assuming a 5% significance level and 80% power.

**Table 1:** Sample size with binary DC

Proportion of patients achieving DC in the control group**	**Proportion of patients achieving DC in the treatment group**	**ICC**	Number of clusters needed*
0.5	0.6	0.01	86
		0.05	114
		0.1	148
0.5	0.7	0.01	22
		0.05	28
		0.1	36
0.5	0.8	0.01	10
		0.05	12
		0.1	16

*The exercise uses a 5% significance level at a power of 80%, with 10 patients per cluster as suggested by Project 686 registries.

**The control group proportion of patient achieving DC is based on our current research in Liuyang.

From the table above, we can see that in order to detect a minimum increase in the proportion of patients achieving DC from 0.5 to 0.6, we will need a sample of at least 860 patients, even assuming a liberal ICC of 0.01. However, if we can achieve a minimum increase of 0.5 to 0.7, even at a conservative ICC of 0.1, only 360 patients are needed for this increase to be detectable. Assuming a patient drop-out rate of 10%, we arrive at a sample size of 400 patients. Project 686 records indicate that villages in the cluster have an average of 10 patients per village; consequently, 40 villages will be needed to gather a sample of 400 villagers with schizophrenia.

### Sample size with the continuous variable drug compliance score (DCS)

The continuous variable DCS will also be used in our data analysis. Our current research in Liuyang indicates a mean DCR score under Project 686 of 7 out of a total score of 10; for a significance level of 0.05 at a power of 80%, and a conservative ICC of 0.1, 38 villages with a total of 380 individuals will be a sample sufficient to detect an effect as small as an increase of 1 in the DCS score. Given the likelihood of drop-outs, we will include 2 additional villages for a total of 40 containing 400 individuals. Once 40 villages have been selected at random, they will be further randomized into 20 villages in the treatment group and 20 in the control group.

### Intervention – VD model

In the VD model, village doctors will receive training in case-management of villagers with schizophrenia using a protocol developed in the pilot study. The three-day training will feature mental health knowledge, case-management skills, and directly observed therapy (DOT). The format will be tailored to the competencies and expectations of VDs. Family members of patients will receive four hours of psycho-educational coaching on how to support patients and cooperate with the village doctor. VDs will participate in the case management protocol for one year and will receive instruction on antipsychotic therapy from visiting psychiatrists at the time of bimonthly drug dispensing.

Specifics of the VD model are given below.

Per Project 686 protocol, psychiatrists dispense drugs, but provide no other services and do not engage family members in the care of patients. In our VD treatment model, VDs cooperate with Project 686 psychiatrists and also reach out to family members to manage patients and provide the following five services:

1. Develop and maintain case files for every schizophrenia patient.

2. Store and distribute antipsychotics to family members on a weekly basis, or directly observe drug-taking (DOT) at the village clinic on a daily basis.

3. Accompany patients and family members on bimonthly visits to psychiatrists for drug dispensation in order to participate in assessing patients’ mental status and explain treatment plans to patients and their families.

4. Record patients’ medication-taking behavior weekly.

5. Identify signs of relapse in order to provide prompt referral services.

To incentivize VDs’ participation, we have obtained preliminary approval to include VDs’ case management efforts in their annual performance review, and will provide a modest fee for VDs for their time and effort.

### Procedures

Following a pilot study (see Section below), 40 villages (excluding the pilot study villages) will be randomly selected in Liuyang Municipality. We will conduct a baseline survey of patients with schizophrenia in the villages, collecting data on social demographics, compliance rates, clinical characteristics, and outcome measures. Researchers will explain the research to participants and obtain written informed consent from both patients and family member. And after the randomization, VDs and family members of patients will receive training and education in the 12-month case management protocol as noted in Section above. Researchers will visit VDs every month during the first three months of the study and every three months thereafter for the remaining nine months of the study, obtaining feedback and giving follow-up instructions, and recording and monitoring VDs’ case management work via questionnaire.

In the CAU model, control villages will continue to receive bimonthly visits by psychiatric professionals, who will dispense free antipsychotics per existing Project 686 protocol. No other interventions will be introduced.

Follow-up interviews will be conducted with patients in both VD and CAU model villages at 6 months and 12 months into the intervention, collecting the same data as that collected at baseline (see below).

### Instruments

Four types of statistics will be collected at baseline and at 6 months and 12 months into the study:

Socio-demographic characteristics: age, sex, education level, marital status, work, economic status, domestic situation (living alone, with family, or with friends).

Clinical characteristics: years of illness, number of bed days in psychiatric hospital in previous two years.

Compliance: This is our primary dependent variable measure. The binary variable Drug Compliance (DC) will be recorded by interviewing patients and their family members. We define as ‘compliant’ patients who, over the past month, have taken all pills as prescribed; otherwise, patients are recorded as ‘non-compliant.’ We will report 1-month, 3-month, 6-month and 12-month compliance as the proportion of patients achieving compliance over the total number of patients. In addition, a continuous variable Drug Compliance Score (DCS) will be used to measure medication compliance. The DCS score will be measured by dividing the actual number of pills taken by the total number of pills prescribed multiplied by 10 over a given period of time, with scores ranging from 0 (complete non-compliance) to 10 (complete compliance). For example, if a patient over the past month took 20 pills out of 100 prescribed, his compliance will be (20/100) × 10 = 2 for the past month.

Clinical/outcome measures: Four types of measures will be collected.

Symptoms score: Schizophrenia is often described in terms of positive and negative (or deficit) symptoms. Negative symptoms respond less well to medication [20], particularly to the antipsychotics that are typically used for cases in our research. Therefore, we will concentrate on the positive symptoms as these correlate more strongly with medication compliance. The Brief Psychiatric Rating Scale (BPRS) will be used to evaluate positive symptoms. The BPRS is an 18-item rating scale that has been widely used in psychiatric practice and research in China, and has good to excellent reliability and validity.

Social functions: We will use the global assessment of functioning (GAF) scale, in which a score of 0 to100 is converted to a disability weight ([100-GAF score]/100). Patients with a disability weight of 0.40 or greater are classified as moderately to severely disabled.

Quality of life score: We will use the QALY weights developed by Lenert *et al*. [21].

Hospitalization rate: Time to readmission is measured by a product-limit formula. Hospitalization rate is the proportion of patients with schizophrenia who warrant hospital admission. Data on hospitalization will be collected from patient medical records.

Incidence of risky behavior: Risky behaviors in schizophrenia include suicide, self-harm, and wandering. We will collect incidence data from family members of patients as to the number of times the above-mentioned behaviors have occurred in the past year.

## Statistical methods

### Comparison between VD model and CAU model

T, Chi-square and various kinds of regression tests will be used to compare DCR (binary variable), DCS (continuous variable), and hospitalization (continuous variable) and incidence of risky behavior (binary variable) rates between the VD and CAU models. Repeated analyses of variance will be used to compare psychiatric symptoms and social function (continuous variable) scores between the two groups.

We will use a difference-in-difference regression model with one treatment variable and additional regressors as control variables (see below). Insofar as perfect randomization and program execution are rarely achieved, we make use of an equation with a difference-in-difference Ordinary Least Square (OLS) estimator with additional regressors to estimate population true coefficients. The example below uses DCS as the dependent variable.

$$\begin{array}{l} \mathrm{\Delta DC}S_{i}=\beta_{0}+\beta_{1}\mathrm{VDmode}l_{i}+\beta_{2}{\text{SocialEconDemog}}_{i}\\ \;+\beta_{3}{\text{SeverityIllness}}_{i}+\beta_{4}{\text{FamilyCare}}_{i}+u_{i} \end{array}$$

∆DCS: This is the value of DCSi for the i^th^ individual at the end of the experiment minus the value of DCS_i_ at the start, or the change. DCS is the Drug Compliance Score, a continuous variable ranging from 0-10, with 0 indicating zero compliance and 10 full compliance.

Treatment (VDmodel): This is a binary variable indicating whether an individual is enrolled in the treatment model (VD) or the control group (CAU). Treatment = 1 if an individual is in the treatment group and 0 for individuals in the control.

Social EconDemog: This refers to social, economic and demographic characteristics that can be plugged into the model as additional control variables for analysis.

Illness Severity: This is a composite of variables relating to illness severity such as number of years an individual has been diagnosed with schizophrenia before the start of our program, and days of hospitalization due to schizophrenia over the two years before the start of the program.

β4FamilyCare i: This variable indicates the level of care an individual receives from his or her family.

u_i_: This is the residual of all other determinants of DCS.

In the above equation, β_1_ indicates program effect, *i.e*., the effectiveness of village doctors in managing villagers with schizophrenia as measured by the average change in DCS for those in the treatment group (VD) minus the average change in DCS for those in the control group (CAU).

There are several advantages to using this model. First, the coefficient in which we are most interested, β_1_, is more efficient than other estimators as it has a smaller variance or standard error than a simple difference estimator when unobserved determinants of DCS persist over time for a given individual. Second, this model eliminates possible pre-program differences in DCS between the treatment and control groups; for example, while using cluster randomization, we may not achieve true randomization at the individual level since it is possible (though unlikely) that all eligible individuals within a cluster could be assigned to a treatment or to a control group. Finally, the difference-in-difference equation allows for additional variables to be added to the treatment variable in the VD group. As these added regressors are possible determinants of the dependent variable DCS, including them improves the efficiency of the OLS estimator, β_1,_ which is the coefficient for program effect, thus addressing: potential bias introduced by imperfect randomization; partial compliance wherein patients with more severe illness in the VD group may at times elect not to comply with village doctors’ care, while patients with milder disease or more supportive family members in the CAU group may, of their own initiative, seek care from their village doctors; and attrition as severely ill patients are lost to follow-up (depending on the data collected, we will seek instrumental variables to address bias introduced by drop-outs). That said, we anticipate a high take-up rate by patients in rural Liuyang because the free antipsychotics program has been operating in the municipality for several years and has been well accepted by patients and their families. Given past experience in Liuyang, we anticipate a drop-out rate of 10% over the course of the study.

### Cost effectiveness analysis

Addressing whether the program is cost-effective is also important. Our cost-utility analysis will assess the cost per quality-adjusted life-years (QALY) added with the program. The analysis is from the societal perspective such that any cost charged to any stakeholder is counted, including volunteer time. The lower the cost per QALY, the more cost-effective the program is. In the present study, costs for treating schizophrenia and controlling symptoms, *e.g*. VD and family members’ time as well as medication costs, will be weighed against averted costs, *e.g*. medical costs of hospitalization and the social costs incurred by the disruption of family and village life by untreated patients. The QUALY costs – and savings – of the proposed study can then be weighed relative to other medical interventions.

We will estimate QALY using a Markov Chain approach, largely modifying and following Garcia-Ruiz *et al*. [22]. As in Garcia-Ruiz, our model will allow for relapses into schizophrenia while taking medication, discontinuing medication due to side effects, or discontinuing medication due to other reasons.

### Pilot study

A pilot study will be conducted in two villages (A and B) before the main project.

### Structured training course

During the pilot study, we will develop a three-day training course for VDs and a four-hour research study and treatment familiarization course for families of patients with schizophrenia. These initiatives will build on our previous experience in developing training courses and will also enlist expert consultation. The training courses will be implemented in the two pilot study villages to be selected by PI, matching the two villages with as much similar characteristics as possible, and feedback from experts and trainees will be collected for continuous quality improvement.

### Methods for distributing antipsychotics

In village A, the VD will distribute antipsychotics to family members on a weekly basis for two months. In village B, patients will receive their medicine in the village clinic every day for DOT. These medication distribution methods will be alternated in villages A and B after two months, so that the total time during which medication distribution is facilitated by VDs will be four months. Feedback on these distribution methods will be collected from participating patients, family members, and VDs in structured focus group discussions. The more feasible distribution method will be determined based on these structured discussions.

### Case-management protocol

A preliminary case-management protocol for VDs will be implemented in villages A and B for four months. The protocol for VD management of patients with schizophrenia will be finalized following the structured discussions described above.

### Reliability and validity of instruments

The reliability and validity of instruments to be used in the baseline survey and in follow-up investigations will be examined during the pilot study. Reliability measures will include test-retest reliability and internal consistency. Validity measures will include structural validity, differences among patients with different age and educational levels, and changes observed consequent to VD case management.

### Sample size validation

Assumptions in calculating sample size will be tested using data collected in the pilot. We will adjust our final sample size accordingly and as necessary.

**Figure 2:**
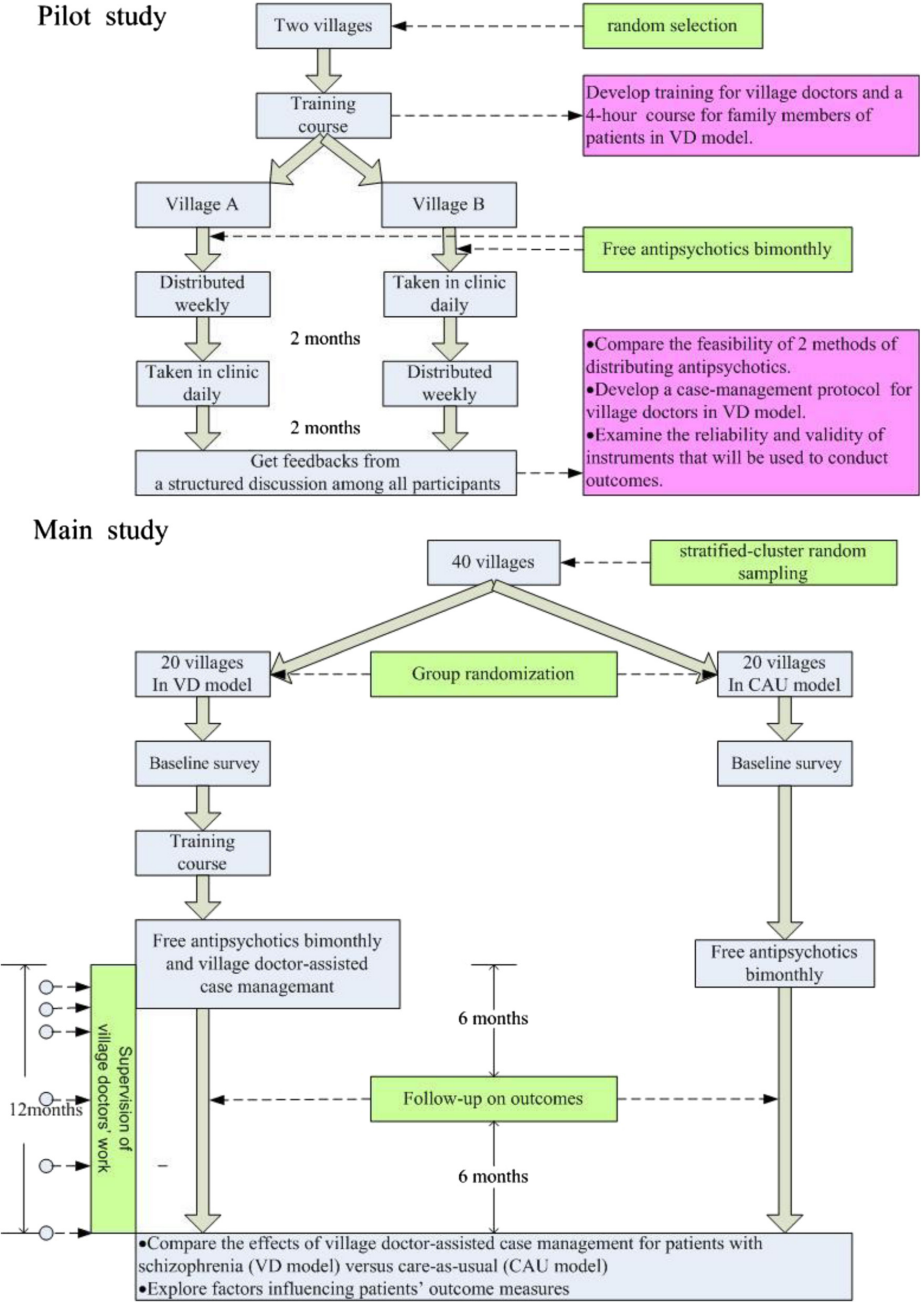
Main activities of pilots and main project.

Figure 2 below summaries the main activities for the pilot and main studies.

### Trial status

The trial started its pilot phase in Sankou and Gugang townships of Liuyang Municipality in July 2013. We are currently in the process of collecting the data from the pilot study.

## Discussion

This randomized controlled study of the role of VDs in managing severely ill mental health patients is the first of its kind in China. We anticipate that VDs living and practicing close to the community, despite their relatively limited medical training, will prove to be valuable assets in improving mental healthcare in low resource areas. Furthermore, this research will help define the role of alternative healthcare workers and lay persons more generally in managing chronic illnesses in areas where access to specialist care is either difficult or unavailable. As this problem is not unique to China, lessons gleaned from this study will likely extend to populations around the globe.

Previous research has suggested various means to improve drug compliance [23]:

1. Empowering patients by involving them in treatment decisions.

2. Improving supervision of patients through a multi-disciplinary team approach.

3. Keeping drug regimes simple and giving close attention to side effects.

4. Improving patient education on medication and side effects, and involving family and friends of the patient in treatment and compliance.

5. Attending to the logistical and administrative aspects of treatment by reducing waiting time, selecting treatment locations preferred by the patient, arranging transportation, and so forth.

Our intervention was designed with these means in mind, with VDs playing a pivotal role in facilitating cooperation among patients, family members, and psychiatrists, while themselves providing essential services in educating patients and their families, supervising treatment (particularly with respect to patients’ drug taking) and monitoring for signs of relapse and other developments requiring referral. Living and working in the same village as patients and their families, VDs are easily accessible to patients and likely have already established a bond of trust. With proper training in case management, their current role as providers of basic medical and preventive care can be expanded to include patient education in, and cooperative treatment and monitoring of, mental illness, thus extending the reach of psychiatry into the rural environment. While our analysis is not sufficiently fine-grained to detect the effect of the various individual strategies listed above on drug compliance, it will deliver substantial data on how VDs function in delivering, coordinating and facilitating a case management protocol in which these strategies are mobilized to improve patient outcomes.

The strength of the proposed RCT is its potential to provide high level evidence on the role of VDs in managing schizophrenia, but the proposal has limitations. Our chief concern has been documenting compliance which, when self-reported by patients, is problematic insofar as patients may be reluctant to report poor compliance. With VDs counting pills, directly observing drug-taking and corroborating compliance with family members, we expect that our study will more likely assess the true treatment situation. A further limitation is that our project piggy-backs on the existing free antipsychotic medication program in Liuyang Municipality; thus, the results may not be fully generalizable to populations in communities where Project 686 has not been implemented. Finally, we must recognize that the Chinese healthcare system has yet to compensate VDs financially for the extra effort involved in providing mental health services. We are hopeful that our research will provide evidence of the public health benefit and cost-effectiveness of employing VDs in the management of serious mental health problems, which may in turn facilitate policy changes in creating system incentives for care-givers who provide these services.

## Abbreviations

ACT: Assertive community treatment; BPRS: Brief psychiatric rating scale; CAU: Care-as-usual; CM: Case management; CRT: Cluster randomized trial; DALY: Disability-adjusted life year; DC: Drug compliance; DCR: Drug compliance score (DCR); DOC: Directly observed therapy; GAF: Global assessment of functioning; ICC: Intra-cluster correlation coefficient; ICM: Intensive case management; LHC: Lay health counselor; LMIC: Low-and middle-income country; MHW: Mental health workers; QoL: Quality of life; RCT: Randomized controlled trial; VD: Village doctors; YLD: Years lived with disability.

## Competing interests

The authors declare that they have no competing interests.

## Authors’ contributions

As the project’s principle investigator, WJG takes primary responsibility in the development and implementation of the project and developed the first draft of this manuscript. SYX supervises WJG’s work and provided guidance in the conception and design of the project. He also made arrangements with government and local authorities to implement this project. DX commented on the manuscript and revised the section on sample size analysis and regression analytical models. LZ contributed to the original design of the project and its statistical methods. HSB developed the cost-effectiveness analysis. KLS, while participating actively in the project development, proofread and refined the entire manuscript. All authors read and approved the final manuscript.

## Authors’ information

Dr. Wenjie Gong is a faculty member of the School of Public Health (SPH) of Central South University (CSU) and is also the principle investigator of this research project supported by the China Medical Board (CMB), which is a U.S. independent foundation providing grants to Chinese medical institutions for almost the past 100 years. Dr. Gong was awarded this grant after a highly competitive open completion for research projects in health policy and systems areas in 2012. Dr. Gong has been a clinical doctor and is currently near her final years in her PhD studies in social medicine while also serving as a faculty member. Dr. Shuiyuan Xiao, a leading public health psychiatrist in China, heads the SPH of CSU. Dr. Xiao also supervises Dr. Gong’s PhD studies. Dr. Xiao has conducted numerous mental health researches, particularly in relation to public health. He is also an opinion leader influencing the mental health policy-making in China. He also leads a collaborative program supported by the CMB in mental health policy studies. Mr. Dong Xu, a PhD student at the Global Health program of University of Washington, was a health policy analyst with training at Harvard University with a master’s degree in public policy. Dr. Liang Zhou, a psychiatrist turned public health researcher at SPH of CSU, has a considerable amount of clinical experiences in mental health and field experiences in conducting mental health experiments. Dr. H. Shelton Brown III, is an economist and an associate professor in management, policy and community health at the University of Texas. He is experienced in cost-effectiveness analysis. Dr. Kirk L. Smith is the Arnold P. Gold Associate Professor of Medicine and a Distinguished Teaching Professor at the University of Texas Medical Branch. In addition to his medical degree, he has a doctorate in Medical Humanities.
